# Complete chloroplast genome sequence of historical olive (*Olea europaea* subsp. *europaea*) cultivar Mehras, in Jordan

**DOI:** 10.1080/23802359.2020.1860712

**Published:** 2021-01-19

**Authors:** Nizar Haddad, Hussein Migdadi, Mohammad Brake, Salam Ayoub, Wisam Obeidat, Yahya Abusini, Abeer Aburumman, Banan Al-Shagour, Eman Al-Anasweh, Monther Sadder

**Affiliations:** a National Agricultural Research Center – NARC, Amman, Jordan; b College of Food and Agricultural Sciences, King Saud University, Riyadh, Saudi Arabia; c Science Faculty, Jerash University, Jerash, Jordan; d School of Agriculture, The University of Jordan, Amman, Jordan

**Keywords:** Mehras, olive, plastome

## Abstract

The complete chloroplast genome sequence of *Olea europaea* subsp. *europaea* cultivar Mehras was determined using high-throughput sequencing technology. Chloroplast genome was 155,897 bp in length, containing a pair of 25,742 bp inverted repeat (IR) regions, which were separated by large and small single-copy regions (LSC and SSC) of 86,622 and 17,791 bp, respectively. The chloroplast genome contained 130 genes (85 protein-coding, 37 tRNA, and eight rRNA). GC content was 37.8%. We performed phylogenetic analysis with other isolates. The analysis showed that *O. e.* subsp. *europaea* cultivar Mehras has an ancient common ancestor with cultivated olives in Italy, Spain, and Cyprus.

Olives are major economic trees grown for both healthy oil and fruits (Besnard et al. 2011; Unver et al. 2017). The oil producing olive cultivar Mehras from Jordan is a historical cultivar, with actively producing fruit trees reaching 1000 year-old (personal communication). Several chloroplast genomes were identified and investigated from *Olea* spp. adapted to different habitats (Besnard et al. 2011; Niu et al. 2020). In this study, we assembled the complete chloroplast genome in Mehras using next generation sequencing. This is part of big project aims to sequence Mehras genome.

Mehras leaves were collected from Alhashemya (Ajloun, Jordan) (32.365906N, 35.663445E). DNA was extracted using total genome wizard kit (Promega, Madison, WI). Library construction of 64 bp pair-end reads and sequencing were carried out using Illumina platform (San Diego, CA). We performed assembling of chloroplast genome by CLC Genomics Workbench (Redwood City, CA) by mapping to reference *O. e.* subsp. *europaea*, isolate Haut Atlas 1 from Morocco (NC_015401 same as FN997650) (Besnard et al. 2011; NCBI 2020) and obtained one contig. Chloroplast annotations were performed from NC_015401 *O. e.* subsp. *europaea* sequence. The chloroplast phylogenetic analysis was performed using previously published chloroplast genome sequences of nine *O. e.* subsp. *europaea* from different regions around the Mediterranean basin and two other species as out-groups (NCBI 2020). Chloroplast sequences were aligned using the multiple alignment (CLC Genomics Workbench, Redwood City, CA). Aligned sequences were used to generate 1000 replicates using the SEQBOOT function available in PHYLIP (Felsenstein 1989). Bootstrapped data were subjected to the maximum likelihood and a consensus tree was generated (Figure 1).

**Figure 1.**
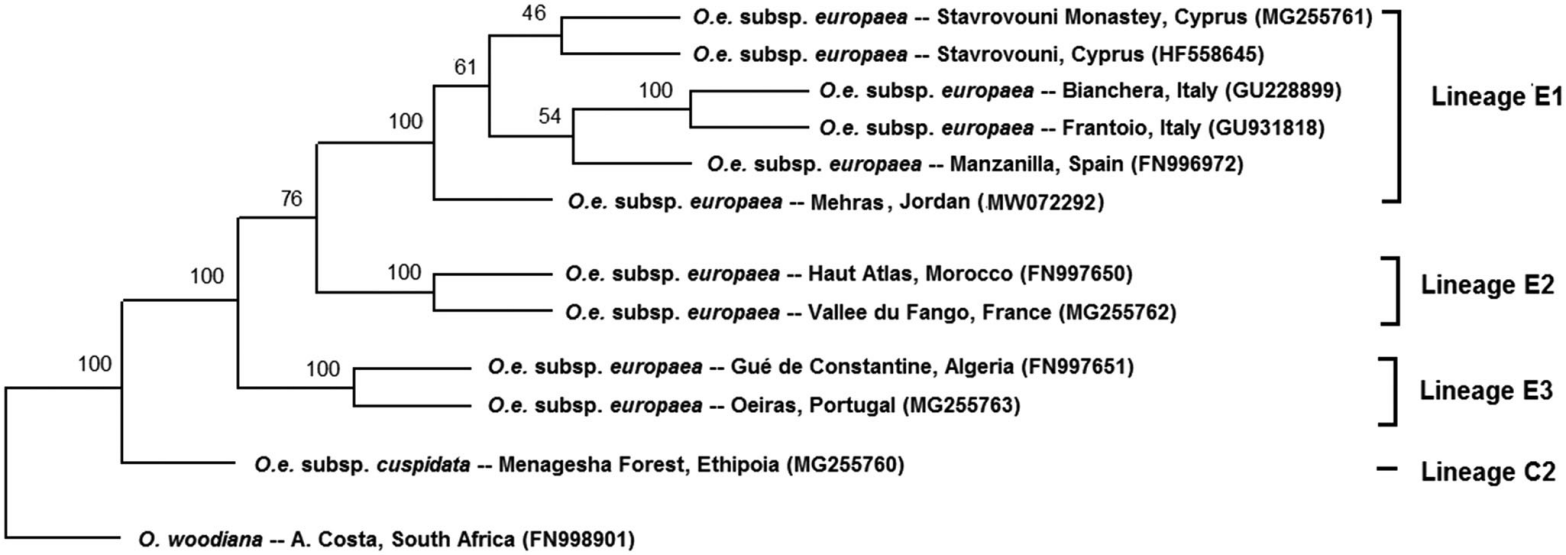
Plastid DNA maximum-likelihood phylogenetic tree of *Olea europaea* subsp. *europaea* cultivar Mehras along with other isolates. Bootstrap values are given on each branch (1000 replicates). *O. e.* subsp. *cuspidata* and *O. woodiana* were used as out-groups. Olive plastid lineages were based on Besnard et al. (2011).

The complement chloroplast genome of Mehras was 155,897 bp length of circular form including four typical regions of chloroplast; large single-copy (LSC) region of 86,622 bp and small single-copy (SSC) region of 17,791 bp, separated by a pair of inverted repeats of 25,742 (IRa and IRb). The chloroplast genome contained 85 protein-coding, 37 tRNA, and eight rRNA genes. The overall GC content was 37.8%. A total of 64 polymorphic loci were found in Mehras chloroplast genome when compared with the *O. e.* subsp. *europaea* reference (NC_015401), they include 11 insertions and eight deletions and 45 SNPs. Furthermore, Mehras chloroplast genome was subject to all 62 polymorphic loci used in classification of *Olea* spp. chloroplast genome lineages (Besnard et al. 2011), where it best aligned with the haplotype E1-1 with two major changes; locus 11 has the 138 bp allele instead of the 126 bp allele and the locus 51 has the 146 bp allele instead of 145 bp allele. The phylogenetic tree showed that *O. e.* subsp. *europaea* cultivar Mehras has a common ancestor with a group of isolates from Italy, Spain and Cyprus, and consequently, this reflects its historical origin when compared to newly developed olive cultivars and provides additional genomic resources for *Olea* spp. studies. For hundreds of years, historic Mehras was adapted to relatively drought environment as compared to other Mediterranean regions producing high quality extra virgin oil. Moreover, revealed unique SNPs as compared with other genotypes from around the Mediterranean (Besnard et al. 2011), makes it a potential genetic resource for olive breeding for improving abiotic stress tolerance.

## Data Availability

The data that support the findings of this study are openly available in GenBank (https://www.ncbi.nlm.nih.gov/) with accession number MW072292 and raw data with accession numbers SRX9347605–SRX9347608.
